# A predictive toxicogenomics signature to classify genotoxic versus non-genotoxic chemicals in human TK6 cells

**DOI:** 10.1016/j.dib.2015.08.013

**Published:** 2015-08-24

**Authors:** Andrew Williams, Julie K. Buick, Ivy Moffat, Carol D. Swartz, Leslie Recio, Daniel R. Hyduke, Heng-Hong Li, Albert J. Fornace, Jiri Aubrecht, Carole L. Yauk

**Affiliations:** a Environmental Health Science and Research Bureau, Health Canada, Ottawa, Ontario, Canada K1A 0K9; b Water and Air Quality Bureau, Health Canada, Ottawa, Ontario, Canada K1A 0K9; c Integrated Laboratory Systems Inc., Research Triangle Park, NC 27709, USA; d Biological Engineering Department, Utah State University, Logan, UT 84322, USA; e Department of Biochemistry and Molecular and Cellular Biology, and Department of Oncology, Georgetown University Medical Center, Washington, District of Columbia 20057, USA; f Drug Safety Research and Development, Pfizer Inc., Groton, CT 06340, USA

## Abstract

Genotoxicity testing is a critical component of chemical assessment. The use of integrated approaches in genetic toxicology, including the incorporation of gene expression data to determine the DNA damage response pathways involved in response, is becoming more common. In companion papers previously published in Environmental and Molecular Mutagenesis, Li et al. (2015) [6] developed a dose optimization protocol that was based on evaluating expression changes in several well-characterized stress-response genes using quantitative real-time PCR in human lymphoblastoid TK6 cells in culture. This optimization approach was applied to the analysis of TK6 cells exposed to one of 14 genotoxic or 14 non-genotoxic agents, with sampling 4 h post-exposure. Microarray-based transcriptomic analyses were then used to develop a classifier for genotoxicity using the nearest shrunken centroids method. A panel of 65 genes was identified that could accurately classify toxicants as genotoxic or non-genotoxic. In Buick et al. (2015) [1], the utility of the biomarker for chemicals that require metabolic activation was evaluated. In this study, TK6 cells were exposed to increasing doses of four chemicals (two genotoxic that require metabolic activation and two non-genotoxic chemicals) in the presence of rat liver S9 to demonstrate that S9 does not impair the ability to classify genotoxicity using this genomic biomarker in TK6cells.

**Specifications Table**

**Table:** 

Subject area	Biology
More specific subject area	Toxicogenomics
Type of data	Genomic Data
How data was acquired	Microarray
Data format	Raw: TXT files; normalized data: TXT files
Experimental factors	TK6 cells, a human lymphoblastoid cell line were obtained from American Type Culture Collection (ATCC# CRL-8015; ATCC, Manassas, VA, USA). Briefly, cells were cultured and maintained in RPMI 1640 medium containing 10% heat inactivated horse serum, in addition to 0.1% pluronics, sodium pyruvate and antibiotics (penicillin at 20 units/ml and streptomycin at 20 µg/ml) at 37±1 °C and 6±1% CO_2_ in air. Immediately prior to chemical exposure, cells were seeded at a density of 4 (±0.5)× 10^5^ cells/ml in twelve-well plates with a final volume of 3 ml per well. For chemicals requiring metabolic activation, exposures were conducted in the presence of 1% 5,6 benzoflavone-/phenobarbital-induced rat liver S9 (BF/PB-induced S9) (Moltox, Boone, NC, USA) with NADPH generating system cofactors.
Experimental features	Transcriptome measurements were performed using a two-color dye swap design [4].
Data source location	Washington, D.C., USA and Ottawa, Ontario, Canada
Data accessibility	National Centre for Biotechnology Information (NCBI) Gene Expression Omnibus (GEO) database Accession: GSE58431 and GSE51175
http://www.ncbi.nlm.nih.gov/geo/query/acc.cgi?acc=GSE58431
http://www.ncbi.nlm.nih.gov/geo/query/acc.cgi?acc=GSE51175

Value of the data

**Table:** 

• The data were integral in developing a genomic biomarker-based approach to predict genotoxicity in human TK6 cells based on expression profiles induced by 28 chemicals that span a variety of well-defined genotoxic and non-genotoxic modes of action. • The data also demonstrate that the use of S9 does not alter gene expression changes used to classify genotoxicity in TK6 cells, expanding on the test agents applied using the biomarker. • The biomarker and these original training data sets can serve as a basis for testing new chemicals for genotoxicity using DNA microarray and other genomics platforms, in other cell types, and potentially in alternative organisms. • The database can be expanded to develop signatures that delve into more detailed genotoxic modes of action (e.g., signatures for cross-linking agents or other types of DNA lesions represented in the training set). • We anticipate that the importance of toxicogenomics studies in chemical risk assessment will continue to increase in the coming years and believe that the rate at which this occurs will be highly dependent upon ensuring public availability of these very powerful datasets sets and tools such as those described.

## 1 Data

The training set (GSE58431) consists of transcriptional changes in TK6 cells following exposure to a diverse set of model agents that include: DNA alkylators, DNA strand breaking agents, topoisomerase inhibitors, nucleotide antimetabolites, endoplasmic reticulum (ER) stressors, energy metabolism inhibitors, histone deacetylase (HDAC) inhibitors, microtubule inhibitors, and heavy metals. From these data, a panel of 65 genes was identified that could accurately classify toxicants as genotoxic or non-genotoxic.

As TK6 cells are not metabolically competent, the test data (GSE51175) examine the utility of the biomarker for use with chemicals requiring metabolic activation in order to broaden the biomarker׳s application [1]. Here, chemical exposures were conducted in the presence of rat liver S9.

## 2 Experimental design, materials and methods

### 2.1 Experimental design

**Fig. 1:**
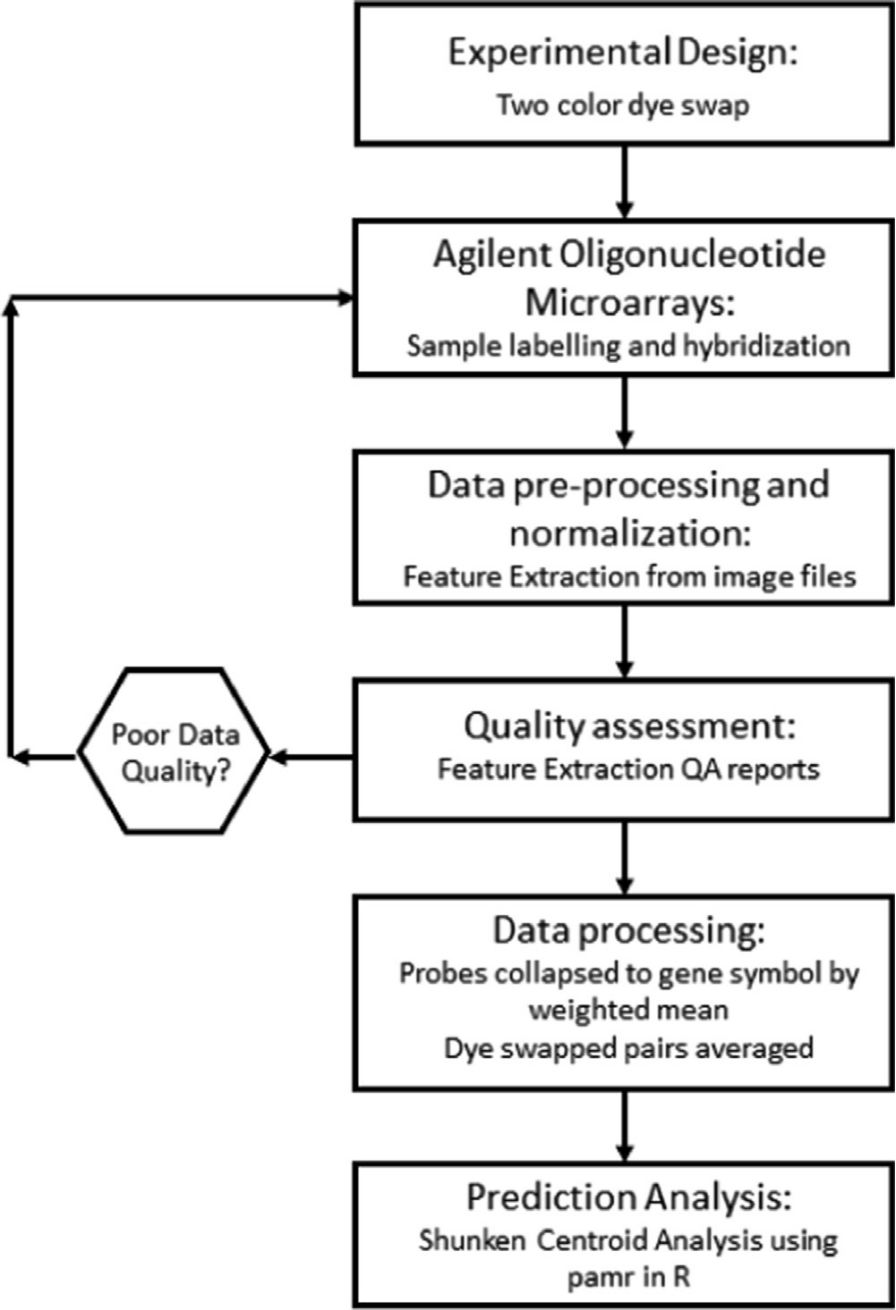
Summary of steps taken to generate, normalize, and analyze two-color, Agilent microarray data.

Transcriptome measurements were performed using a two-color dye swap design [4]. For each agent, cyanine-3 and cyanine-5 labeled cRNA for treated and untreated samples were co-hybridized to two Agilent oligonucleotide microarrays. One microarray was hybridized with the treated cyanine-3 labeled cRNA co-hybridized with the untreated cyanine-5 labeled cRNA. The second slide was hybridized with the treated and untreated samples having the reversed cyanine-3 and cyanine-5 labeling. A summary of the workflow for the subsequent steps are presented as Fig. 1.

### 2.2 Hybridization and data quantification

Hybridization and washing was performed according to the manufacturer׳s protocol. Arrays were scanned with an Agilent DNA microarray scanner. Feature Extraction (Version 9.1; Agilent) was used to quantify the scanned images for the 4x44k platform, to calculate the processed signal intensity and to estimate the log ratios (log 10). Similarly for the 8x60k platform, Agilent Feature Extraction software (version 11.0.1.1) was used for quantification of the generated image files, to generate the QC reports as well as the processed signal intensity and to estimate the log ratios (log 10).

### 2.3 Data processing and normalization

Microarray annotation files for the Agilent human 1x44k (eArray 1x44K human_20120130.txt), 4x44k (eArray 4x44K human_20120130.txt), 4x44k version 2 (eArray 4x44K human_v2_20120130.txt), 8x60k (eArray 8x60K human_20120411.txt), and the 8x60k version 2 (eArray 8x60K human_v2_20120628.txt) platform were obtained from eArray (https://earray.chem.agilent.com/earray/). These annotation files were read into the R statistical environment [6]. All unique Agilent probe ids from these platforms were used to create an annotation file using the Probes.R script named Agilent annotation.txt. This annotation file was then merged with the Feature Extraction data files to collapse the probe ID׳s to the gene symbol.

Agilent Feature Extraction.TXT files were read into the R environment using the Read.R script. This R script extracts the normalized relative intensities, merges the Agilent annotation.txt file using the probe ids and uses the weighted mean to average the probes with multiple gene symbols. Once all the data files have been processed, the individual data sets were then merged together using the gene symbol. The data were merged together such that the dye swaps were in adjacent columns. The dye swaps were then averaged and the processed data file named LogRatio.txt was written out to disk.

### 2.4 Development of the TGx-28.65 genotoxicity classifier

The original 65-gene signature, as published in Li et al. [5], referred to as TGx-28.65 (28 refers to the use of 28 chemicals in the training set) was developed using the nearest shrunken centroids approach [7]. Gene expression data were exported from Rosetta Resolver based on Entrez Gene Identifiers. The data were then read into the R statistical environment employing the pamr package [3]. The standardized centroids to predict whether an agent is genotoxic or not (either directly or indirectly) was computed for each class using a training set of agents, as described by Li et al. Briefly, the standardized centroid is the mean expression level for each gene in a class divided by its within-class standard deviation. The standard centroid for each class is then shrunken toward the overall centroid to produce the nearest shrunken centroid. The method employs a shrinkage parameter that is used to control the number of features used to construct the classifier.

The shrinkage parameter was identified by employing 10-fold cross validation [2]. This is done by randomly assigning samples to one of ten approximately equal-sized sets, which is also roughly balanced for the two classes. Prediction accuracy is assessed for each set with the other nine sets used to construct a classifier. From this analysis a shrinkage threshold of 2.2 produced a 65-gene panel with 100% accuracy based on 10-fold cross-validation.

### 2.5 Application of the TGx-28.65 signature to TK6 cells co-exposed with rat liver S9

Due to revisions in the gene annotation and differences between the 4x44k and 8x60k platforms, two of the 65 genes in the TGx-28.65 biomarker were unavailable when the +S9 data were incorporated into the study. As a result, MGC5370 (due to the annotation update) and USP41 (not present on the 8x60k platform) were removed from the TGx-28.65 biomarker.

Using the remaining 63 genes, the centroids were re-estimated using the PAM.R script. This R script reads in the LogRatio.txt data into the R statistical environment. Using the training set, the pamr package was used to update the classifier. These were written to disk using the filename Classifier.txt. The summarized data are presented in Table 1. Removing MGC5370 and USP41 did not impact the performance of the classifier as the accuracy remained 100% (estimated using 10-fold cross-validation).

### 2.6 PCA analysis

**Fig. 2:**
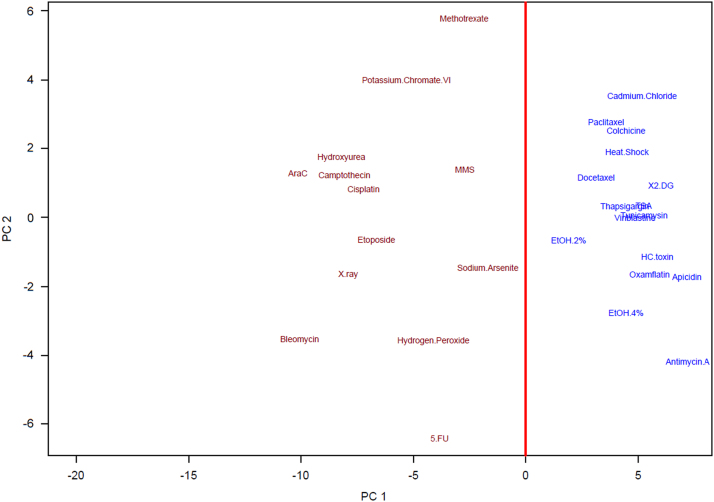
Scatter plot of the first and second principle component of the TGx-28.65 training set. The vertical red line indicates the first principle component at 0. The font for the genotoxic agents is red and the font for the non-genotoxic agents is blue.

A principle component analysis (PCA) was performed using the prcomp function [8] in R on the training set data (Li et al. data) from the LogRatio.txt data file. Using the 63 genes in the TGx-28.65 classifier the PCA was conducted using the PCA.R script. A summary of the PCA results are presented in Table 2 and the scatter plot of the first two principle components for the training set are presented as Fig. 2. The red line in Fig. 2 was added as the first principle component and could be interpreted as a contrast between the genotoxic (red) and non-genotoxic (blue) classes as there is a clear separation between the two classes.

**Fig. 3:**
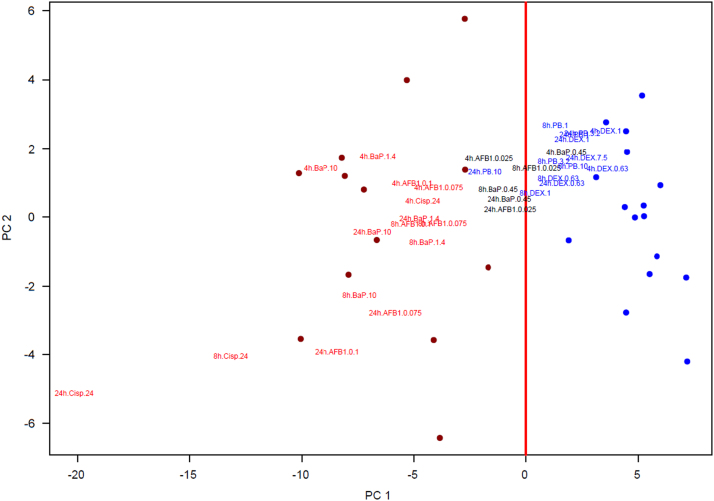
Scatter plot of the first and second principle component of the TGx-28.65 training set with the +S9 data. The vertical red line indicates the first principle component at 0. The genotoxic agents from the training set are represented by red circles and the non-genotoxic agents with blue circles. The font for the genotoxic agents with S9 are displayed in red font and the non-genotoxic agents with S9 are presented using the blue font.

The PCA loadings obtained from this analysis was applied to the +S9 data. A scatter plot of these data for the first two principle components is presented as Fig. 3. The data from the training set are represented by the red and blue circles, whereas the labels for the +S9 data are displayed with red font for the genotoxic agents and blue font for the non-genotoxic agents. For the +S9 data, the low dose samples for the genotoxic agents were displayed in gray as their genotoxicity was uncertain. Similar to Fig. 2, the genotoxic agents and non-genotoxic agents separate into two groups with the low dose genotoxic agents falling in the middle.

## Conflicts of interest

None.

## Figures and Tables

**Table 1:** TGx-28.65 classifier. Presented are the estimated standard deviation and the shrunken centroids for the genotoxic and non-gentoxic classes.

ID	Standard deviation	Centroids		ID	Standard deviation	Centroids	
		Genotoxic-score	Non-genotoxic score			Genotoxic score	Non-genotoxic score
ACTA2	0.198	0.390	−0.330	HIST1H3D	0.244	−0.324	0.274
AEN	0.217	0.895	−0.758	ID2	0.275	−0.512	0.433
ARRDC4	0.270	0.554	−0.469	IKBIP	0.198	0.625	−0.529
B3GNT2	0.243	−0.513	0.434	ITPKC	0.170	0.460	−0.389
BLOC1S2	0.182	0.907	−0.768	ITPR1	0.233	−0.573	0.484
BRMS1L	0.219	0.509	−0.431	LCE1E	0.312	0.704	−0.595
BTG2	0.207	0.854	−0.722	LRRFIP2	0.204	−0.515	0.436
C12orf5	0.225	0.635	−0.537	MDM2	0.197	0.807	−0.683
CBLB	0.183	−0.617	0.522	MEX3B	0.218	0.457	−0.386
CCP110	0.175	0.638	−0.540	NLRX1	0.208	0.469	−0.397
CDKN1A	0.243	0.702	−0.594	PCDH8	0.259	0.829	−0.701
CEBPD	0.328	0.502	−0.425	PHLDA3	0.211	1.026	−0.868
CENPE	0.203	−0.546	0.462	PLK3	0.256	0.468	−0.396
COIL	0.273	0.425	−0.359	PPM1D	0.176	1.131	−0.957
DAAM1	0.258	−0.570	0.482	PRKAB1	0.206	1.106	−0.936
DCP1B	0.161	0.763	−0.646	PRKAB2	0.195	0.499	−0.422
DDB2	0.181	0.872	−0.738	PTGER4	0.260	−0.605	0.512
DUSP14	0.181	0.498	−0.421	RAPGEF2	0.226	−0.577	0.488
E2F7	0.190	0.943	−0.798	RBM12B	0.182	0.521	−0.441
E2F8	0.237	0.670	−0.567	RPS27L	0.183	0.557	−0.471
EI24	0.174	0.545	−0.461	RRM2B	0.272	0.601	−0.508
FAM123B	0.224	0.608	−0.514	SEL1L	0.242	−0.301	0.255
FBXO22	0.158	0.695	−0.588	SEMG2	0.234	0.414	−0.351
GADD45A	0.243	0.646	−0.546	SERTAD1	0.253	0.927	−0.785
GXYLT1	0.157	0.368	−0.311	SMAD5	0.211	0.500	−0.423
HIST1H1E	0.311	−0.471	0.398	TM7SF3	0.174	0.607	−0.514
HIST1H2BB	0.326	−0.276	0.234	TNFRSF17	0.357	0.550	−0.466
HIST1H2BC	0.329	−0.318	0.269	TOPORS	0.236	0.482	−0.408
HIST1H2BG	0.349	−0.325	0.275	TP53I3	0.198	0.703	−0.595
HIST1H2BI	0.333	−0.263	0.222	TRIAP1	0.232	0.912	−0.772
HIST1H2BM	0.356	−0.275	0.233	TRIM22	0.234	0.847	−0.717
HIST1H2BN	0.219	−0.218	0.185				

**Table 2:** Summary of the PCA results. The standard deviation, proportion of variance and the cumulative proportion of variance is presented for the first nine principle components. The standard deviations for the other principle components not presented were less than 1.

	PC1	PC2	PC3	PC4	PC5	PC6	PC7	PC8	PC9
Standard deviation	5.9363	2.6398	2.23332	1.74326	1.49113	1.39276	1.16271	1.02611	1.01938
Proportion of variance	0.5594	0.1106	0.07917	0.04824	0.03529	0.03079	0.02146	0.01671	0.01649
Cumulative proportion	0.5594	0.6700	0.74914	0.79738	0.83267	0.86346	0.88492	0.90163	0.91813

## References

[1] Buick J.K., Moffat I., Williams A., Swartz C.D., Recio L., Hyduke D.R., Li H.H., Fornace A.J., Aubrecht J., Yauk C.L. (2015). Integration of metabolic activation with a predictive toxicogenomics signature to classify genotoxic versus nongenotoxic chemicals in human TK6 cells. Environ Mol Mutagen.

[2] Hastie T., Tibshirani R., Friedman J.H. (2001). The Elements of Statistical Learning: Data Mining, Inference, and Prediction (With 200 Full-Color Illustrations).

[3] Hastie, T., Tibshirani, R., Narasimhan, B., & Chu, G. pamr: Pam: prediction analysis for microarrays. R package version 1.54.1, 〈http://CRAN.R-project.org/package=pamr〉, 2013.

[4] Kerr M.K., Churchill G.A. (2001). Statistical design and the analysis of gene expression microarray data. Genet. Res..

[5] Li H.H., Hyduke D.R., Chen R., Heard P., Yauk C.L., Aubrecht J., Fornace A.J. (2015). Development of a toxicogenomics signature for genotoxicity using a dose-optimization and informatics strategy in human cells. Environ. Mol. Mutagen..

[6] R-Core-Development-Team (2012). R: A language and environment for statistical computing.

[7] Tibshirani R., Hastie T., Narasimhan B., Chu G. (2002). Diagnosis of multiple cancer types by shrunken centroids of gene expression. Proc. Natl. Acad. Sci. USA.

[8] Venables W.N., Ripley B.D. (2002). Modern Applied Statistics with S.

