# Supplementary material for: A predictive toxicogenomics signature to classify genotoxic versus non-genotoxic chemicals in human TK6 cells
Source: Data Brief. 2015 Aug 24;5:77–83. doi: 10.1016/j.dib.2015.08.013 (PMC4564388; doi:10.1016/j.dib.2015.08.013)
Supplement: Supplementary file 2 — Supplementary data [file mmc2.doc]

Conflicts of interest: none
